# Adjusting for verification bias in diagnostic accuracy measures when comparing multiple screening tests - an application to the IP1-PROSTAGRAM study

**DOI:** 10.1186/s12874-021-01481-w

**Published:** 2022-03-18

**Authors:** Emily Day, David Eldred-Evans, A. Toby Prevost, Hashim U. Ahmed, Francesca Fiorentino

**Affiliations:** 1 Imperial Clinical Trials Unit, Imperial College London, London, UK; 2 Imperial Prostate, Division of Surgery, Department of Surgery and Cancer, Faculty of Medicine, Imperial College London, London, UK; 3 Nightingale-Saunders Unit, King’s Clinical Trials Unit, King’s College London, London, UK; 4 Imperial Urology, Imperial College Healthcare NHS Trust, London, UK; 5 Division of Surgery, Imperial College London, St Mary’s Hospital, Praed Street, London, W2 1NY UK

**Keywords:** Verification bias, Sensitivity, Specificity, Multiple imputation, Begg and Greenes

## Abstract

**Introduction:**

Novel screening tests used to detect a target condition are compared against either a reference standard or other existing screening methods. However, as it is not always possible to apply the reference standard on the whole population under study, verification bias is introduced. Statistical methods exist to adjust estimates to account for this bias. We extend common methods to adjust for verification bias when multiple tests are compared to a reference standard using data from a prospective double blind screening study for prostate cancer.

**Methods:**

Begg and Greenes method and multiple imputation are extended to include the results of multiple screening tests which determine condition verification status. These two methods are compared to the complete case analysis using the IP1-PROSTAGRAM study data. IP1-PROSTAGRAM used a paired-cohort double-blind design to evaluate the use of imaging as alternative tests to screen for prostate cancer, compared to a blood test called prostate specific antigen (PSA). Participants with positive imaging (index) and/or PSA (control) underwent a prostate biopsy (reference standard).

**Results:**

When comparing complete case results to Begg and Greenes and methods of multiple imputation there is a statistically significant increase in the specificity estimates for all screening tests. Sensitivity estimates remained similar across the methods, with completely overlapping 95% confidence intervals. Negative predictive value (NPV) estimates were higher when adjusting for verification bias, compared to complete case analysis, even though the 95% confidence intervals overlap. Positive predictive value (PPV) estimates were similar across all methods.

**Conclusion:**

Statistical methods are required to adjust for verification bias in accuracy estimates of screening tests. Expanding Begg and Greenes method to include multiple screening tests can be computationally intensive, hence multiple imputation is recommended, especially as it can be modified for low prevalence of the target condition.

**Supplementary Information:**

The online version contains supplementary material available at 10.1186/s12874-021-01481-w.

## Introduction

Screening trials are conducted to evaluate the success of a novel screening test method to detect the target condition, when compared against the reference (“gold”) standard method or other existing screening methods of detecting the target condition [1].

It is not always possible to carry out the reference standard on the whole population under study. The invasiveness or high cost of certain types of reference standard testing make them impractical or unethical for use on the whole population. Therefore, screening tests that are less invasive, and less expensive than the reference standard methods used in clinical practice can be first employed (screening test) to determine which participants should undergo the definitive test (reference standard).

Binary screening tests yield either screen-positive or screen-negative results for detecting the condition of interest and a binary reference standard indicates presence or absence of the target condition. A reference standard method is assumed to yield perfect or near-perfect detection; in reality many reference tests are the best test that one can use. A screen-positive result on the initial screening test would usually imply that a participant would undergo further condition verification (by the reference standard). This implies that condition verification is more likely to be conducted in individuals with a positive screening test result, and verification is more likely to be absent for cases with a negative screening test result. This introduces verification bias in the diagnostic evaluation of a suitable screening test. In this paper, we will focus only on binary screening tests.

Accuracy measures such as sensitivity, specificity, negative predictive value (NPV) and positive predictive value (PPV) express how well screening tests under evaluation are able to identify participants as having the target condition [2]. Calculating the sensitivity and specificity estimates, based only on those cases who have undergone condition verification, overestimates the sensitivity and underestimates the specificity of the screening test [3], due to the lack of information for participants who received a negative screening test result.

The unadjusted approach to tackle this type of problem is the “complete case analysis” approach [4]. However, this does not correct for verification bias, hence sensitivity estimates may be inflated, and specificity estimates may be deflated [5]. Several statistical methods have been proposed to correct for verification bias encountered in the design of this type of screening trial. The most commonly used methods are the one developed by Begg and Greenes [5] and multiple imputation [6]. Begg and Greenes proposed a method which relies on the key assumption that the chance of undergoing the reference standard depends only on observed variables (i.e. the screening test results) and not directly on unobserved condition status [5] – similar to the missing at random (MAR) assumption [7]. Empirical methods such as bootstrapping are commonly employed to estimate confidence intervals for accuracy estimates calculated by this method. The other common approach is to treat the condition status of the non-verified participants as a missing data problem and implement a multiple imputation algorithm [8] to impute these missing condition statuses. This flexible approach allows for the inclusion of multiple diagnostic tests, as well as prognostic factors which are known to predict condition status, under the MAR assumption [7].

There are various examples in the literature of using Begg and Greenes to adjust for verification bias when verification status only depends on a single screening test [9–11]. There are few examples of using this method and applying it to two screening tests that determine participant verification status [4]. When searching the literature, we could not find evidence of these methods being used for more than two screening tests.

The aim of this paper is to extend the Begg and Greenes method and the multiple imputation algorithm to adjust accuracy measures to account for verification bias, when verification depends on three independent screening tests. We use the data collected during the IP1-PROSTAGRAM study [12] to demonstrate the use of these methods when three screening tests are used, independently and in no prescriptive order, to determine whether a participant should undergo condition verification or not and compare it to complete case analysis (where no adjustment for verification bias is done). We compare accuracy measures (sensitivity, specificity, PPV and NPV) for each of the three screening tests, along with their corresponding 95% confidence intervals, between the complete case method (unadjusted approach), Begg and Greenes method, and multiple imputation.

## Material and methods

### The clinical study: IP1-PROSTAGRAM

IP1-PROSTAGRAM [12] was a prospective, blinded, population-based screening study for prostate cancer, conducted from October 2018 to August 2019. The novel screening methods were Magnetic Resonance Imaging (MRI) and shearwave ultrasound which were used in parallel with traditional serum Prostate Specific Antigen (PSA) (an existing screening test for prostate cancer). Participants underwent all three screening tests in no prescriptive order. If any one of the three screening test results were positive, participants were advised to undergo a biopsy for histological verification (reference standard) (see Appendix 1). The aim was for each patient to undergo all screening tests with the results of each test blinded. Operators of each screening test were blinded to the results of the other screening tests. Participants with positive results were informed that one or more test results were positive but not informed which test result was positive until study completion. All participants were unblinded on study completion, including those who tested negative on all screening tests [12]. Condition status, defined as presence or absence of clinically significant prostate cancer, was determined by verification on biopsy. Additionally, and separately to the radiologist, a Computer Aided Detection (CAD) system was used for reading the MRI results for all patients. The primary definition, of general acceptance [13], for clinically significant prostate cancer was Gleason ≥3 + 4 (Grade Group (GrG) ≥ 2) and is used in the IP1-PROSTAGRAM study application of the methods to adjust for verification bias. The study specific screen-positive thresholds which determined condition verification for the three screening tests were MRI (PIRADS [Prostate Imaging-Reporting and Data System]/ Likert) ≥ 3, ultrasound (US [Ultrasound Score] scoring system) ≥ 3 and PSA level ≥ 3.0 ng/ml.

IP1-PROSTAGRAM recruited 408 participants, of whom 403 had complete results for all screening tests (MRI, ultrasound and PSA). Five participants were excluded from this analysis as 3 were missing ultrasound results and 2 were missing MRI results. One hundred sixty-five patients had a positive result from at least one of the screening tests and went on to undergo a confirmatory biopsy for clinically significant prostate cancer. One patient, who had all three negative screening tests, underwent a confirmatory biopsy for clinically significant prostate cancer because the CAD system identified a lesion which met the criteria to warrant a biopsy. Hence 166/403 (41.2%) patients underwent a confirmatory biopsy.

Table 1 outlines the composition of the 237 participants who did not undergo condition verification in terms of screening test result combinations. The majority of these participants had three negative screening test results (220/237; 92.8%). Seventeen participants had at least one positive screening test result but withdrew from the study and so never underwent a biopsy (non-verified). Baseline demographics for these participants, who withdrew from the study (and so did not undergo a biopsy (non-verified)), were similar to the baseline demographics of the participants who had at least one positive screening test result and who did not withdraw (and so underwent a confirmatory biopsy (verified)). This is plausibly because participants were blinded to their screening test results up until withdrawal.

**Table 1:** Screening test results for participants who did not undergo condition verification in the IP1-PROSTAGRAM trial

Verification Status	MRI	Ultrasound	PSA	Total
***V*** **= 0**	0	0	0	**220**
	1	0	0	5
	0	1	0	5
	0	0	1	1
	1	1	0	3
	1	0	1	1
	0	1	1	2
	1	1	1	0
	**Total**			**237**

^1^MRI/ultrasound/PSA = 1: screen-positive result

^2^MRI/ultrasound/PSA = 0: screen-negative result

^3^*V* = 0: did not undergo condition verification (reference standard)

### Methods notation

*R*, *S* and *T* are screening tests which can be represented collectively in a vector ***Q*** *=* (*R, S, T*), where:

- *R* = 1*, S* = 1*, T* = 1 if the result is screen-positive for screening tests *R*, *S*, *T*, respectively*,*
- *R* = 0*, S* = 0*, T* = 0 if the result is screen-negative for screening tests *R*, *S*, *T*, respectively.

*V* is a participant's verification status:

- *V* = 1 if the participant has undergone the reference standard,
- *V* = 0 if the participant has not undergone the reference standard.

*D* is a participant's condition status:

- *D* = 1 if the participant has the target condition (according to the reference standard result),
- *D* = 0 if the participant does not have the target condition (according to the reference standard result).

### Definitions

For the following definitions, it is assumed that the target condition status is operationalised by the reference standard result.

Sensitivity is the ability of a screening test, say (*R*), to correctly identify those participants who have the target condition (*D* = 1). That is, the probability that the screening test result is screen-positive, e.g. *R* = 1, given that the participant has the target condition (*D* = 1), hence sensitivity of *R* = Pr(*R* = 1| *D* = 1). This would be calculated as the proportion of participants who have a screen-positive screening test result and have the target condition out of the total number of participants who have the target condition, if there were no missing data.

Specificity is the ability of a screening test, say (*R*), to correctly identify those participants who do not have the target condition (*D* = 0). That is, the probability that the screening test result is screen-negative, e.g. *R* = 0, given that the participant does not have the target condition (*D* = 0), hence specificity of *R* = Pr(*R* = 0| *D* = 0). This would be calculated as the proportion of participants who have a screen-negative screening test result and do not have the target condition out of the total number of participants who do not have the target condition, if there were no missing data.

PPV is the probability that the participant has the target condition (*D* = 1), given they had a screen-positive screening test result. So for test *R* we have (*R* = 1): PPV = Pr(*D* = 1| *R* = 1). This would be calculated as the proportion of participants who have a screen-positive screening test result and have the target condition out of the total number of participants who have a screen-positive screening test result, if there were no missing data.

NPV is the probability that the participant does not have the target condition (*D* = 0), given they had a screen-negative screening test result. For test *R* (*R* = 0): NPV = Pr(*D* = 0| *R* = 0). This would be calculated as the proportion of participants who have a screen-negative screening test result and do not have the target condition out of the total number of participants who have a screen-negative screening test result, if there were no missing data.

### Methods to deal with verification bias

The following statistical methods to account for verification bias in the calculation of accuracy measure estimates can be implemented when condition verification is dependent on the results of multiple screening tests.

#### Complete case analysis (unadjusted approach)

Using this method, only participants who underwent the reference standard (verified) and have complete screening test and reference standard results are included in the analysis and all non-verified participants are omitted from the analysis. Accuracy measures are calculated using data from those participants who underwent condition verification, and so have complete screening test results and reference standard data. 95% confidence intervals for each of the accuracy measures are computed in the standard way [14].

#### Begg and Greenes for multiple screening tests

The application of Begg and Greenes for one screening test is already described [4, 5, 15]. This application has been extended to include two screening tests [4]. We will focus here on describing the application to three screening tests.

Begg and Greenes method uses observed proportions of those who have and do not have the target condition among the verified participants to calculate the expected number of those who have and do not have the target condition among those participants who did not undergo condition verification [4]. They proposed an empirical method [5] to correct for verification bias when there are incomplete data on condition status for those who had not undergone verification. This method [5] assumes that the prevalence of the target condition estimated in the subset of participants who are screen-negative and undergo verification applies to all screen-negatives [16]. By design, this assumption does not hold when all screen-negative participants do not undergo verification for the target disease. Hence, it is recommended, in practice, that a randomly selected proportion of participants with screen-negative results undergo verification by the reference standard [17].

The method relies on the MAR assumption [7]. When applied to multiple screening tests, this assumption implies that within the strata of the combinations of the screening tests the distribution of participants is random and uses this logic to then compute the accuracy measures. Under the MAR assumption, verification status (*V*) and condition status (*D*) are conditionally independent on observed variables [5]. That is, whether or not a participant undergoes the reference standard is not determined by the true condition status of the participant, but instead is conditional on observed variables, such as screening test results. Due to the assumed independence of *V* and *D* (MAR assumption), it follows that Pr(*V*| ***Q***) = Pr(*V*| ***Q***, *D*). From this, it also follows, that Pr(*D*| ***Q***) = Pr(*D*| ***Q***, *V*) and Pr(*D*| ***Q***) = Pr(*D*| ***Q***, *V* = 1), where *V* = 1 represents undergoing condition verification.

Then, by Bayes theorem for screening test *R* [4]:

$$\Pr \left(R|D,S,T\right)=\frac{\sum_S{\sum}_T\Pr \left(\boldsymbol{Q}\right)\Pr \left(D|\boldsymbol{Q},V=1\right)}{\sum_{\boldsymbol{Q}}\Pr \left(\boldsymbol{Q}\right)\Pr \left(D|\boldsymbol{Q},V=1\right)}$$

where Pr(***Q***) and Pr(*D*| ***Q***, *V* = 1) can be directly estimated from the data.

De Groot et al. [4] tabulated Begg and Greenes method for two screening tests. Verified and non-verified participant proportions are combined to create a completed “two-by-two” table as if all participants had received the reference standard. We tabulate (Table 2) the Begg and Greenes method for three screening tests that, combined, determine condition verification.

**Table 2:** Begg and Greenes method for three screening tests (*R, S, T*)

	Screening Tests^**1**^			Condition Status^**3**^		
Verification Status^**2**^	***R***	***S***	***T***	***D*** = 1	***D*** = 0	Total
***V*** **= 1**	0	0	0	*a* ^6^	*b*	*T1_000*^4^
	1	0	0	*c*	*d*	*T1_100*
	0	1	0	*e*	*f*	*T1_010*
	0	0	1	*g*	*h*	*T1_001*
	1	1	0	*i*	*j*	*T1_110*
	1	0	1	*k*	*l*	*T1_101*
	0	1	1	*m*	*n*	*T1_011*
	1	1	1	*o*	*p*	*T1_111*
***V*** **= 0**	0	0	0	*a’* ^7^	*b’*	*T0_000*^5^
	1	0	0	*c’*	*d’*	*T0_100*
	0	1	0	*e’*	*f’*	*T0_010*
	0	0	1	*g’*	*h’*	*T0_001*
	1	1	0	*i’*	*j’*	*T0_110*
	1	0	1	*k’*	*l’*	*T0_101*
	0	1	1	*m’*	*n’*	*T0_011*
	1	1	1	*o’*	*p’*	*T0_111*

^1^Three screening tests *R, S, T*:

Screen-positive result: *R* = 1*, T* = 1*,* and/or *S* = 1

Screen-negative result: *R* = 0, *T* = 0, and *S* = 0

^2^*V* – Verification status:

*V* = 1 for verified participants (those who underwent the reference standard)

*V* = 0 for non-verified participants (those who did not undergo the reference standard).

^3^*D* – Condition status:

*D* = 1 for those who have the target condition (according to the reference standard result)

*D* = 0 for those who do not have the target condition (according to the reference standard result).

^4^*T1_000, T1_100, T1_010, T1_001, T1_110, T1_101, T1_011, T1_111* are the total numbers of verified (*V* = 1) participants with each combination of screening test results. These totals can be found from the data. To satisfy the assumption that the prevalence of the target condition estimated in the subset of participants who are screen-negative and undergo verification applies to all screen-negatives, *T1_000* > 0 must hold

^5^*T0_000, T0_100, T0_010, T0_001, T0_110, T0_101, T0_011, T0_111* are the total numbers of non-verified (*V* = 0) participants with each combination of screening test results. These totals can be found from the data

^6^*a, b, c, d, e, f, g, h, I, j, k, i, m, n, o, p* are the numbers of verified participants (*V* = 1) with each of the combinations of screening test results, with (*D* = 1) or without (*D* = 0) the target condition. These frequencies are known from the data

^7^*a’, b’, c’, d’, e’, f’, g’, h’, i’, j’, k’, l’, m’, n’, o’, p’* are numbers of non-verified participants (*V* = 0) with each of the combinations of screening test results, with (*D* = 1) or without (*D* = 0) the target condition. These frequencies are missing from the data, but can be estimated from the known values for the verified patients and the total numbers of non-verified patients with each combination of the screening test results

The MAR assumption [7] assumes that participants with a specific combination of screening test results who have not been verified would have shown a similar distribution of condition status, which is proportional, to those with the same specific combination of screening test results who were verified. By this, the number of non-verified participants with each combination of screening test results in Table 2 can be calculated:

$${a}^{\prime }=\frac{a}{a+b}\times T0\_000$$

$${b}^{\prime }=\frac{b}{a+b}\times T0\_000$$

$${c}^{\prime }=\frac{c}{c+d}\times T0\_100$$

$${d}^{\prime }=\frac{d}{c+d}\times T0\_100$$

$${e}^{\prime }=\frac{e}{e+f}\times T0\_010$$

$${f}^{\prime }=\frac{f}{e+f}\times T0\_010$$

$${g}^{\prime }=\frac{g}{g+h}\times T0\_001$$

$${h}^{\prime }=\frac{h}{g+h}\times T0\_001$$

$${i}^{\prime }=\frac{i}{i+j}\times T0\_110$$

$${j}^{\prime }=\frac{j}{i+j}\times T0\_110$$

$${k}^{\prime }=\frac{k}{k+l}\times T0\_101$$

$${l}^{\prime }=\frac{l}{k+l}\times T0\_101$$

$${m}^{\prime }=\frac{m}{m+n}\times T0\_011$$

$${n}^{\prime }=\frac{n}{m+n}\times T0\_011$$

$${o}^{\prime }=\frac{o}{o+p}\times T0\_111$$

$${p}^{\prime }=\frac{p}{o+p}\times T0\_111$$

Then, combining Bayes theorem for, say, screening test *R*, the MAR assumption, and Table 2 frequency estimates, we can calculate the accuracy estimates for screening test *R*, where condition status (*D*) is operationalised by the result of the reference standard, and adjusting for verification bias we get:

$$\mathrm{Sensitivity}=\Pr \left(R=1|D=1,S,T\right)=\frac{c+{c}^{\prime }+i+{i}^{\prime }+k+{k}^{\prime }+o+o^{\prime }}{a+{a}^{\prime }+c+{c}^{\prime }+e+{e}^{\prime }+g+{g}^{\prime }+i+{i}^{\prime }+k+{k}^{\prime }+m+{m}^{\prime }+o+o^{\prime }},$$

$$\mathrm{Specificity}=\Pr \left(R=0|D=0,S,T\right)=\frac{b+{b}^{\prime }+f+{f}^{\prime }+h+{h}^{\prime }+n+n^{\prime }}{b+{b}^{\prime }+d+{d}^{\prime }+f+{f}^{\prime }+h+{h}^{\prime }+j+{j}^{\prime }+l+{l}^{\prime }+n+{n}^{\prime }+p+p^{\prime }},$$

$$\mathrm{PPV}=\Pr \left(D=1|R=1,S,T\right)=\frac{c+{c}^{\prime }+i+{i}^{\prime }+k+{k}^{\prime }+o+o^{\prime }}{c+{c}^{\prime }+d+{d}^{\prime }+i+{i}^{\prime }+j+{j}^{\prime }+k+{k}^{\prime }+l+{l}^{\prime }+o+{o}^{\prime }+p+p^{\prime }},$$

$$\mathrm{NPV}=\Pr \left(D=0|R=0,S,T\right)=\frac{b+{b}^{\prime }+f+{f}^{\prime }+h+{h}^{\prime }+n+n^{\prime }}{a+{a}^{\prime }+b+{b}^{\prime }+e+{e}^{\prime }+f+{f}^{\prime }+g+{g}^{\prime }+h+{h}^{\prime }+m+{m}^{\prime }+n+n^{\prime }}.$$

Similarly, it is possible to calculate the accuracy measures for screening tests *S* and *T* (Appendix 2). Bootstrapping [18] can be used to estimate the confidence intervals for the Begg and Greenes accuracy estimates [16].

#### Multiple imputation for multiple screening tests

Verification bias can be considered as a missing data problem [6]. Due to verification bias, the condition status for those participants who did not undergo verification is missing. By using a multiple imputation method, it is possible to impute the missing condition status, based on the results of the screening tests and the verified condition status [6, 8, 19]. A multiple imputation algorithm to impute missing condition status in non-verified participants [8] can be applied to adjust accuracy measures for verification bias. First, the probability of recommendation for condition verification by reference standard depends on the results of the screening tests.

Imputation of missing condition status is conducted following the steps below:

1. Verification status is dependent on the results of the screening tests. To account for this, a logistic regression model is fitted for condition status (dichotomised *D*) on *n* dichotomised screening test results (*X*_*i*_; *i* = 1, . . , *n*), for the subset of participants who underwent condition verification (*V* = 1) where the fitted logistic regression model coefficients are defined as (*β*_*i*_; *i* = 1, . . , *n*).
2. For each non-verified participant (who did not undergo the reference standard, *V* = 0), the individual probability of having the target condition (*D* = 1) is estimated based on the screening test results (*X*_*i*_; *i* = 1, . . , *n*), and the coefficients (*β*_*i*_; *i* = 1, . . , *n*) from the fitted logistic regression model, using the inverse logistic function, and a random binary variable (0/1) will be drawn with this probability using the uniform distribution. This imputes the missing condition status for the non-verified participants.
3. Accuracy measures (sensitivity, specificity, PPV, NPV) for each screening test are calculated, along with their 95% confidence intervals, for the complete screening population using the imputed condition statuses for non-verified participants (*V* = 0), and recorded true condition statuses for verified participants (*V* = 1).
4. This process is repeated for *m* iterations, due to the implementation of the uniform distribution using the calculated predicted probability to impute the missing condition statuses for those participants who did not undergo the reference standard (*V* = 0). For each iteration the calculated test accuracy estimates and their 95% confidence intervals are stored.
5. The verification bias adjusted estimates are the mean values of all estimates from the *m* iterations.
6. 95% confidence intervals are combined using Rubin’s rules [7], accounting for variation within and between the imputed datasets.

#### Multiple imputation for the IP1-PROSTAGRAM results

In IP1-PROSTAGRAM, the number of dichotomised screening tests is *n* =3. It is important to consider the prevalence of positive results that are verified. In order to use binary logistic regression for the multiple imputation method the assumption based on the widely adopted minimal guideline criterion for sample size considerations of 10 events per variables (EPV) [20–22] included in the model need to be considered. This is because logit coefficients suffer from small-sample bias [23, 24], leading to systematically overestimated associations. The estimation of logit coefficients by maximum likelihood is sometimes inaccurate when EPV is low. Firth’s correction [25] is a general approach to reducing small-sample bias in maximum likelihood estimation. Firth’s correction adds a penalty on the likelihood which removes a portion of the small-sample bias anticipated by the maximum likelihood method. The penalty will tend to zero as the sample size increases [26]. Firth’s correction has been shown to reduce finite sample bias close to zero and reduce mean square error. Using simulation studies [26], it has been shown that the performance of logistic regression can be significantly improved using Firth’s correction when EPV is low.

Since in the IP1-PROSTAGRAM study the number of positive verified results was less than 10 per variable (dichotomised screening test result) Firth’s correction was used in the logistic regression. Moreover, due to the small number of patients with clinically significant prostate cancer, we computed Wilson Score 95% confidence intervals using the method derived by Lott and Reiter [27], extending Rubin’s rules [7] to combine Wilson Score intervals after multiple imputation. To compute the 95% confidence intervals for sensitivity and specificity we used an effective sample size, as introduced and used by Li, Mehrotra and Barnard [28].

## Results

Using the IP1-PROSTAGRAM study data [12], accuracy measures were calculated using the study specific screen-positive thresholds which determined disease verification.

The complete case analysis only uses data for participants who underwent a confirmatory biopsy and have complete screening test results and histology results, hence it uses data for 166 participants (166/403, 41.2%).

Begg and Greenes and multiple imputation methods use data for all participants who had complete screening test results data whether they underwent a confirmatory biopsy or not (*N* = 403).

For Begg and Greenes all the different combinations of screening tests results were considered. Since we have results for three binary (screen-positive vs screen-negative) screening tests which determined verification status then we have 8 different combinations of screening test results (Table 2). For IP1-PROSTAGRAM, we assumed that *R* = MRI, *S* = ultrasound, *T* = PSA, *D* is the presence (*D* = 1) or absence (*D* = 0) of clinically significant cancer as determined by biopsy (reference standard) results, and *V* is whether a participant underwent biopsy (*V* = 1) or did not (*V* = 0). The number of participants in each category for IP1-PROSTAGRAM is estimated using Table 2 and is outlined in Table 4 (Appendix 3).

Using the multiple imputation method for verification bias adjustment, we verified the independence of the three screening tests by studying the pairwise and three-way interactions in the logistic regression model. None of the interaction terms were statistically significant, and so were not included in the final model. The final model only included main effects for each screening test. We repeated the imputation process for 100 (*m*) iterations.

Since in the IP1-PROSTAGRAM data there are few events of clinically significant cancer (*N* = 16/403), we have also used multiple imputation fitting a penalised logistic regression model (using the same steps 1–6 in the Methods section), with Firth’s correction, to predict prostate cancer status (*D*), from the results of the three screening tests (MRI, ultrasound and PSA) for those participants who underwent a biopsy (*V* = 1). We repeated the imputation process for 100 (*m*) iterations, as in the non-penalised multiple imputation method.

Table 3 presents the accuracy measures for the three screening tests (MRI, ultrasound and PSA) used in the IP1-PROSTAGRAM trial. Also included is the prevalence of positive screening results by each screening test, and prevalence of clinically significant prostate cancer for each of the verification bias adjustment methods.

**Table 3:** Accuracy Measure (Sensitivity, Specificity, PPV and NPV) Estimates, with 95% Confidence Intervals, Adjusted for Verification Bias

			Statistical Methods for Verification Bias Adjustment			
			Complete Case Analysis (Unadjusted Approach) (***N*** = 166)	Begg and Greenes using Three Screening Tests (***N*** = 403)	Multiple Imputation using Three Screening Tests (using standard logistic regression) (***N*** = 403)	Multiple Imputation using Three Screening Tests, using penalised logistic regression (Firth’s method) (N = 403)
		Prevalence of clinically significant prostate cancer	9.6% (5.6 to 15.2%)	4.5% (2.6 to 6.8%)	4.7% (3.0 to 7.3%)	4.0% (2.5 to 6.4%)
**Screening Tests**	**MRI**	**Prevalence of positive MRI**	53.0% (45.4 to 60.5%)	24.1% (20.1 to 28.5%)	24.1% (20.1 to 28.5%)	24.1% (20.1 to 28.5%)
		**Accuracy measures**	Sens = 87.5% (61.7 to 98.4%) Spec = 50.7% (42.4 to 58.9%) PPV = 15.9% (9.0 to 25.2%) NPV = 97.4% (91.0 to 99.7%)	Sens = 87.9% (68.8 to 100.0%) Spec = 78.9% (74.8 to 82.9%) PPV = 16.4% (9.8 to 24.8%) NPV = 99.3% (98.0 to 100.0%)	Sens = 82.2% (58.9 to 88.0%) Spec = 78.8% (74.4 to 82.6%) PPV = 16.0% (10.0 to 24.9%) NPV = 98.9% (97.0 to 99.6%)	Sens = 87.5% (64.0 to 96.5%) Spec = 78.6% (74.2 to 82.4%) PPV = 14.4% (8.8 to 22.8%) NPV = 99.3% (97.6 to 99.8%)
	**US**	**Prevalence of positive ultrasound**	51.2% (43.6 to 58.8%)	23.6% (19.7 to 28.0%)	23.6% (19.7 to 28.0%)	23.6% (19.7 to 28.0%)
		**Accuracy measures**	Sens = 56.3% (29.9 to 80.2%) Spec = 49.3% (41.1 to 57.6%) PPV = 10.6% (5.0 to 19.2%) NPV = 91.4% (83.0 to 96.5%)	Sens = 56.3% (31.7 to 78.3%) Spec = 78.0% (73.8 to 81.6%) PPV = 10.7% (5.2 to 18.1%) NPV = 97.4% (95.3 to 99.0%)	Sens = 53.7% (31.6 to 74.3%) Spec = 77.9% (73.5 to 81.7%) PPV = 10.6% (5.9 to 18.7%) NPV = 97.2% (94.7 to 98.5%)	Sens = 56.3% (33.2 to 76.9%) Spec = 77.8% (73.4 to 81.6%) PPV = 9.5% (5.1 to 17.0%) NPV = 97.7% (95.4 to 98.9%)
	**PSA**	**Prevalence of positive PSA**	21.1% (15.5 to 28.0%)	9.7% (7.1 to 13.0%)	9.7% (7.1 to 13.0%)	9.7% (7.1 to 13.0%)
		**Accuracy measures**	Sens = 37.5% (15.2 to 64.6%) Spec = 80.7% (73.4 to 86.7%) PPV = 17.1% (6.6 to 33.6%) NPV = 92.4% (86.4 to 96.3%)	Sens = 36.4% (14.2 to 63.8%) Spec = 91.6% (88.5 to 94.0%) PPV = 16.9% (5.4 to 29.3%) NPV = 96.8% (94.5 to 98.4%)	Sens = 35.6% (17.1 to 58.0%) Spec = 91.5% (88.4 to 93.9%) PPV = 16.6% (8.1 to 31.4%) NPV = 96.6% (94.2 to 98.0%)	Sens = 37.5% (18.5 to 61.4%) Spec = 91.5% (88.3 to 93.9%) PPV = 15.4% (7.2 to 29.7%) NPV = 97.3% (95.0 to 98.5%)

Accuracy measure (sensitivity, specificity, PPV and NPV) estimates adjusted for verification bias, using complete cases analysis (unadjusted approach), Begg and Greenes using three screening tests, multiple imputation using three screening tests, and multiple imputation using three screening tests and penalised logistic regression (Firth’s correction) (IP1-PROSTAGRAM trial data [12])

In the complete case analysis, the number of participants with clinically significant prostate cancer was 16 (9.6%; 95% CI: 5.6–15.2%). Taking non-verified participants into account using Begg and Greenes and multiple imputation (using standard logistic regression) inflates these estimates slightly as these methods predict that some non-verified participants would have had clinically significant prostate cancer if they had undergone a confirmatory biopsy, based on their screening test results. For Begg and Greenes, the number of clinically significant prostate cancer cases was 18 (4.5%; 95% CI: 2.6–6.8%). The number of cases of clinically significant prostate cancer using multiple imputation are similar to Begg and Greenes, i.e. 19 (4.7, 95% CI: 3.0–7.3%).

However, using multiple imputation with penalised logistic regression, the number of clinically significant prostate cancer cases remains the same as for complete case analysis, namely 16 (4.0%; 95% CI: 2.5–6.4%). Using multiple imputation with penalised logistic regression did not result in any of the missing condition statuses for non-verified participants being clinically significant prostate cancer.

## Discussion

We extended three commonly used statistical methods to adjust for verification bias when comparing three screening tests with a reference standard, when the reference standard is not always carried out.

We found that sensitivity estimates remained similar across the four methods, with overlapping 95% confidence intervals, with a slight decrease in sensitivity estimates when comparing complete case analysis to Begg and Greenes (for PSA) and multiple imputation using standard logistic regression (for all screening tests). This slight decrease in sensitivity estimates is expected as we know from the literature that sensitivity estimates are inflated when using the data only for those participants who undergo condition verification [5]. Particularly, sensitivity estimates, and 95% confidence intervals, were similar for complete case analysis and multiple imputation using penalised logistic regression. None of the missing condition statuses of the non-verified participants were imputed as having clinically significant prostate cancer, the number of true positives, and those who had the target condition does not change between complete case and multiple imputation using penalised logistic regression. In fact, we did not expect sensitivity to vary dramatically, when adjusting for verification bias, due to the nature of the patient population recruited in the IP1-PROSTAGRAM study [12] being the general population of men with no specific indication of likelihood of having the target condition.

The most noticeable difference is in the specificity estimates. From the literature, we know that specificity estimates are deflated when only considering complete cases [5]. When comparing complete case results to those of Begg and Greenes and both methods of multiple imputation (penalised and standard logistic regression), there is a significant increase in the specificity estimates for all screening tests. This increase in specificity estimates is statistically significant at a 5% significance level, demonstrated by non-overlapping 95% confidence intervals, for all screening tests. The majority of the non-verified participants (Table 3) will contribute to the specificity estimates, rather than the sensitivity estimates by definition of these accuracy measurements. The specificity estimates calculated by Begg and Greenes and both methods of multiple imputation are similar, with almost completely overlapping 95% confidence intervals. Therefore, these methods are supportive of each other.

The point estimates for NPV are higher when using Begg and Greenes and both methods of multiple imputation, compared to complete case analysis. The corresponding 95% confidence intervals around NPV estimates tend to be narrower when adjusting for verification bias, compared to complete case analysis. These narrowed confidence intervals can be explained by the increase in information used in the NPV estimates for Begg and Greenes and both methods of multiple imputation due to the use of the non-verified participants with incomplete data on condition status. The 95% confidence intervals for the NPV estimates compared across methods overlap, implying the difference in NPV estimates is not significant. Comparing PPV estimates and their corresponding 95% confidence intervals highlights the similarities of these estimates between the methods employed to adjust for verification bias. This is supported by the literature [15, 29, 30] which indicates that PPV and NPV are not significantly affected by verification bias and hence reporting PPV and NPV without adjusting for verification bias is acceptable.

In our application of multiple imputation methods to the IP1-PROSTAGRAM data, using penalised logistic regression does not significantly affect the accuracy measure estimates. The point estimates are similar when compared between the two methods of multiple imputation for all screening tests, with almost completely overlapping 95% confidence intervals.

The multiple imputation method could be extended to include baseline prognostic factors that are known to be associated with having clinically significant prostate cancer [6, 8], if justified by the value of EPV [26].

A limitation of Begg and Greenes method [5] is that it can only be applied when a subset of participants who are screen-negative (had negative results on all screening tests) undergo target condition verification [16]. In practice it is recommended that a subset of screen-negative participants undergo verification by the reference standard [17] to avoid any issues with this assumption. In IP1-PROSTAGRAM, patients with all negative screening tests would not have undergone verification of target condition by design. However, one patient in the study underwent disease verification with three negative initial screening tests, and so Begg and Greenes method holds for these data.

### Accuracy of reference standard

For the IP1-PROSTAGRAM study, we considered biopsy to be an accurate reference standard method for detection of clinically significant prostate cancer. The study was not setup to assess the accuracy of the reference standard diagnosis. The study did not conduct repeat biopsies, or long-term follow-up confirmation to understand the degree to which the reference standard is accurate.

### Comparison to other previous published works

In 1998 Zhou X-H [31] reviewed developments in bias-correction methods for studies on the accuracy of diagnostic tests. His paper focuses on developments on maximum likelihood estimators and implementation of Begg and Greenes [5]. He considered the application of methods to a single binary diagnostic test, two correlated binary tests, ordinal diagnostic test and two ordinal-scale diagnostic tests. In 2006 Harel and Zhou [6] demonstrated the use of multiple imputation techniques to handle verification bias in screening trials. The authors introduce the application of different multiple imputation processes to address the problem of incomplete data. They then compare the accuracy estimates and confidence intervals for a single screening test calculated using five multiple imputation methods, and Begg and Greenes [5] using simulated datasets, and real-world examples in liver disease and breast cancer. Later, this analysis was reassessed by De Groot et al. [19], who demonstrate that Begg and Greenes [5] and multiple imputation [6] produce similar results when correcting for verification bias in the context of a single binary screening test. In our paper, we build on these principles, applying these methods to the case of three independent screening tests using data collected prospectively in the IP1-PROSTAGRAM study [12].

Cronin and Vickers [32] use a simulation study to compare the complete case method (unadjusted approach) to Begg and Greenes [5], comparing area under the curve (AUC) statistics, rather than accuracy estimates directly, when varying both the rate and mechanism of verification. They focus on single binary screening tests. They then apply these methods to real world examples in cervical cancer [33] and prostate cancer screening [9], as well as single photon emission computed tomography [34] to compare results of estimates of AUC when using different methods to adjust for verification bias.

De Groot et al. [4] use a large dataset on patients with deep venous thrombosis [35] that underwent condition verification by the reference standard, and set the true condition status to missing based on various underlying mechanisms and a varying total number of missing values. The authors then compare the performance of different bias correction methods to the estimates using the completed dataset. They compare Begg and Greenes [5], using both one and two binary screening tests, and multiple imputation, and demonstrate that the Begg and Greenes and multiple imputation estimates are similar. We have extended these methods to incorporate three independent screening tests which determined whether or not a participant underwent condition verification.

More recently, Xue et al. [16] use weighted estimating equations to investigate the accuracy of multiple screening tests as well as simultaneously compare results between screening tests while addressing verification bias. These equations are used in simulations and a real-world example of cervical cancer screening. This method does not appear to have been as widely used in the literature as Begg and Greenes, and multiple imputation.

We are not currently aware of any evidence of these methods being used for more than two screening tests.

## Conclusions

Specificity and NPV estimates computed by the complete case method are prone to verification bias, and should be adjusted. Sensitivity estimates do not vary dramatically when independent screening tests are carried out which give concordant negative results. All accuracy measure estimates calculated using Begg and Greenes and both methods of multiple imputation are similar for all screening tests. Expanding Begg and Greenes method to include multiple screening tests can be computationally intensive. Since the estimates are similar to those calculated using multiple imputation, this is the preferred method. If EPV is low in the binary outcome variable, penalised logistic regression (Firth’s correction) should be used to improve the performance of the multiple imputation algorithm. If EPV is sufficient, then the multiple imputation algorithm can be expanded to include more screening tests that determine condition verification and prognostic factors that are associated with having the target condition.

## Supplementary Information

**Additional file 1.**

## Data Availability

The datasets analysed during the current study are available from the Chief Investigator of the IP1-PROSTAGRAM study, Professor Hashim U. Ahmed, on reasonable request.

## Abbreviations

AUC: Area under the curve
CAD: Computer Aided Detection
EPV: Events per variable
GrG: Grade Group
MAR: Missing at random
MRI: Magnetic resonance imaging
PIRADS: Prostate Imaging-Reporting and Data System
PPV: Positive predictive value
PSA: Prostate Specific Antigen
NPV: Negative predictive value
US: Ultrasound score
