# Supplementary material for: Adjusting for verification bias in diagnostic accuracy measures when comparing multiple screening tests - an application to the IP1-PROSTAGRAM study
Source: BMC Med Res Methodol. 2022 Mar 18;22:70. doi: 10.1186/s12874-021-01481-w (PMC8932251; doi:10.1186/s12874-021-01481-w)
Supplement: Supplementary file 1 — Additional file 1. [file 12874_2021_1481_MOESM1_ESM.docx]

**Appendices**

1. **
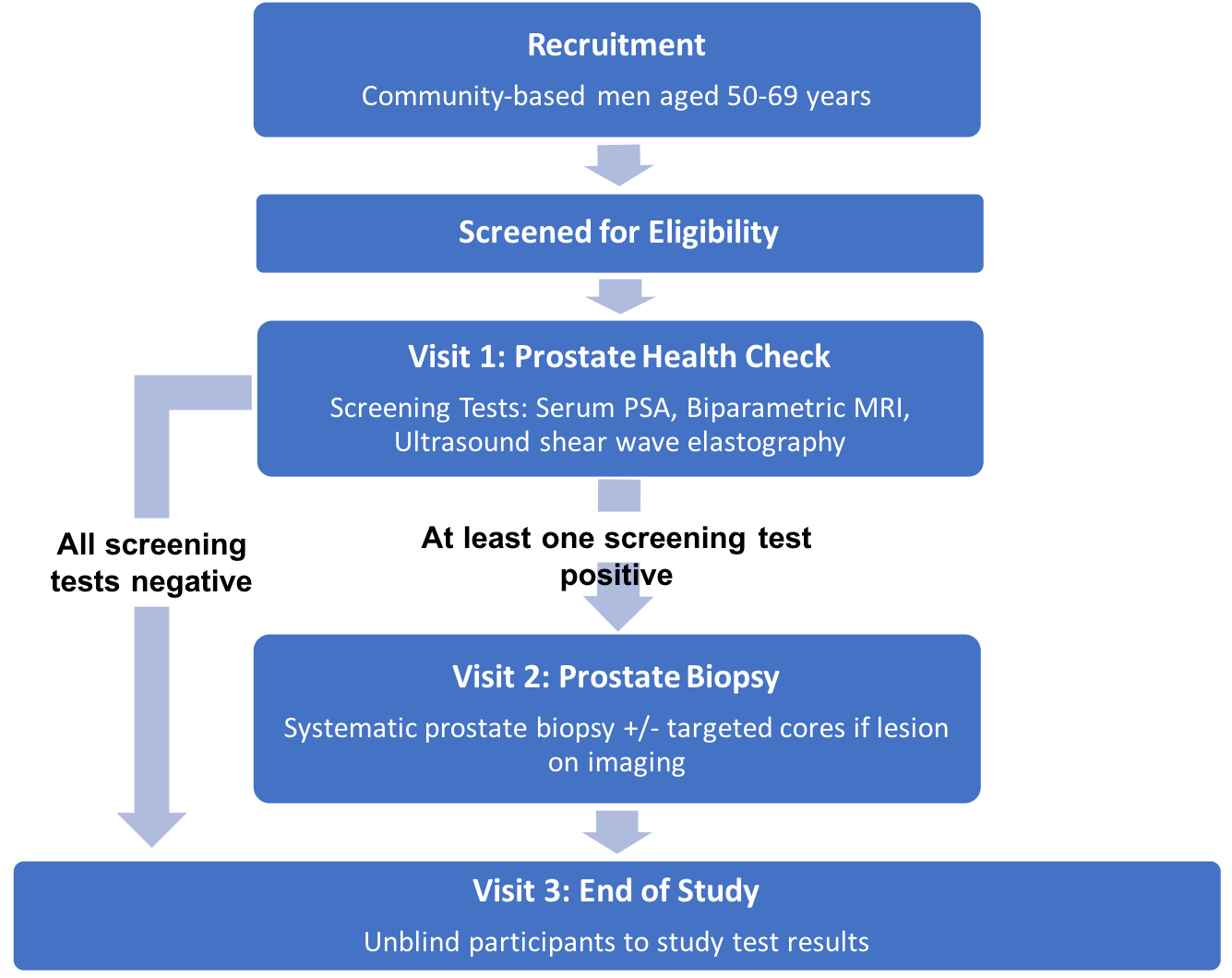
Appendix 1 – IP1-PROSTAGRAM Study**

**Figure 1: IP1-PROSTAGRAM Visit Schedule (adapted from** (12)**)**

1. **Appendix 2 – Accuracy Estimates for Screening Tests *S* and *T***
   1. **Screening Test *S***

Bayes theorem for screening test *S*:

$$\Pr\left( S | D, R, T \right)= \frac{\sum_{R} \sum_{T} \Pr\left( R, S, T \right)Pr(D|R,S,T,V+)}{\sum_{R} \sum_{S} \sum_{T} \Pr\left( R, S, T \right)Pr(D|R, S, T, V+)}$$

Using Bayes theorem (for screening test *S*), the MAR assumption, and Table 2 frequency estimates, we can calculate the accuracy estimates for screening test *S,* adjusting for verification bias:

Sensitivity = $Pr(S=1|D=1, R, T)$

$$=\frac{e+e^{'}+i+i^{'}+m+m^{'}+o+o'}{a+a^{'}+c+c^{'}+e+e^{'}+g+g^{'}+i+i^{'}+k+k^{'}+m+m^{'}+o+o'}$$

Specificity = $Pr(S=0|D=0,R, T)$

$$=\frac{b+b^{'}+d+d^{'}+h+h^{'}+l+l'}{b+b^{'}+d+d^{'}+f+f^{'}+h+h^{'}+j+j^{'}+l+l^{'}+n+n^{'}+p+p'}$$

PPV = $Pr(D=1|S=1, R, T)$

$$=\frac{e+e^{'}+i+i^{'}+m+m^{'}+o+o'}{e+e^{'}+f+f^{'}+i+i^{'}+j+j^{'}+m+m^{'}+n+n^{'}+o+o^{'}+p+p'}$$

NPV = $Pr(D=0|S=0, R, T)$

$$=\frac{b+b^{'}+d+d^{'}+h+h^{'}+l+l'}{a+a^{'}+b+b^{'}+c+c^{'}+d+d^{'}+g+g^{'}+h+h^{'}+k+k^{'}+l+l'}$$

- 1. **Screening Test *T***

Bayes theorem for screening test *T*:

$$\Pr\left( T | D, R, S \right)= \frac{\sum_{R} \sum_{S} \Pr\left( R, S, T \right)Pr(D|R,S,T,V+)}{\sum_{R} \sum_{S} \sum_{T} \Pr\left( R, S, T \right)Pr(D|R, S, T, V+)}$$

Using Bayes theorem (for screening test *T*), the MAR assumption, and Table 2 frequency estimates, we can calculate the accuracy estimates for screening test *T*, adjusting for verification bias:

Sensitivity = $Pr(T=1|D=1, R, S)$

$$=\frac{g+g^{'}+k+k^{'}+m+m^{'}+o+o'}{a+a^{'}+c+c^{'}+e+e^{'}+g+g^{'}+i+i^{'}+k+k^{'}+m+m^{'}+o+o'}$$

Specificity = $Pr(T=0|D=0,R, S)$

$$=\frac{b+b^{'}+d+d^{'}+f+f^{'}+j+j'}{b+b^{'}+d+d^{'}+f+f^{'}+h+h^{'}+j+j^{'}+l+l^{'}+n+n^{'}+p+p'}$$

PPV = $Pr(D=1|T=1, R, S)$

$$=\frac{g+g^{'}+k+k^{'}+m+m^{'}+o+o'}{g+g^{'}+h+h^{'}+k+k^{'}+l+l^{'}+m+m^{'}+n+n^{'}+o+o^{'}+p+p'}$$

NPV = $Pr(D=0|T=0, R, S)$

$$=\frac{b+b^{'}+d+d^{'}+f+f^{'}+j+j'}{a+a^{'}+b+b^{'}+c+c^{'}+d+d^{'}+e+e^{'}+f+f^{'}+i+i^{'}+j+j'}$$

1. **Appendix 3 – Estimates for the Begg and Greenes Method** (4) **using the IP1-PROSTAGRAM study data** (12)

**Table 4: Begg and Greenes method for three screening tests using the IP1-PROSTAGRAM data** (12)

|  | **Screening Tests** | | |  | **Condition Status** | |  |
| --- | --- | --- | --- | --- | --- | --- | --- |
| **Verification Status** | **MRI (≥3)** | **Ultrasound (≥3)** | **PSA (≥3ng/ml)** |  | **Clinically significant cancer (D=1)** | **Absence of clinically significant cancer (D=0)** | **Total** |
| **Underwent biopsy (V=1)** | 0 | 0 | 0 |  | 0 | 1 | 1 |
|  | 1 | 0 | 0 |  | 4 | 60 | 64 |
|  | 0 | 1 | 0 |  | 1 | 51 | 52 |
|  | 0 | 0 | 1 |  | 1 | 11 | 12 |
|  | 1 | 1 | 0 |  | 5 | 9 | 14 |
|  | 1 | 0 | 1 |  | 2 | 2 | 4 |
|  | 0 | 1 | 1 |  | 0 | 13 | 13 |
|  | 1 | 1 | 1 |  | 3 | 3 | 6 |
| **Did not undergo biopsy (V=0)** | 0 | 0 | 0 |  | 0 | 220 | 220 |
|  | 1 | 0 | 0 |  | 0.3125 | 4.6875 | 5 |
|  | 0 | 1 | 0 |  | 0.09615385 | 4.9038462 | 5 |
|  | 0 | 0 | 1 |  | 0.08333333 | 0.91666667 | 1 |
|  | 1 | 1 | 0 |  | 1.0714286 | 1.9285714 | 3 |
|  | 1 | 0 | 1 |  | 0.5 | 0.5 | 1 |
|  | 0 | 1 | 1 |  | 0 | 2 | 2 |
|  | 1 | 1 | 1 |  | 0 | 0 | 0 |

**Begg and Greenes method for three screening tests (*MRI, Ultrasound and PSA*) using the IP1-PROSTAGRAM data** (12)
